# Dynamic Echocardiographic Imaging of a Valve-in-Valve Mitral Prosthesis

**DOI:** 10.1155/2022/1366037

**Published:** 2022-02-16

**Authors:** Bishoy Wassef, Mina Masry, Mounir Ghali, John N. Makaryus, Amgad N. Makaryus

**Affiliations:** ^1^Department of Sports Medicine, RWJBarnabas Health, Care Station Medical Group, Secaucus, NJ, USA; ^2^Department of Cardiology, Nassau University Medical Center, East Meadow, NY, USA; ^3^Department of Pulmonary and Critical Care Medicine, University of Michigan Health, Ann Arbor, MI, USA; ^4^Department of Cardiology, North Shore University Hospital and Donald and Barbara Zucker School of Medicine at Hofstra/Northwell, Hempstead, NY, USA

## Abstract

Dynamic imaging of heart valves and specifically prosthetic valves is a central benefit of echocardiography. Most bioprosthetic heart valves degenerate over a given time and hence require repeat valve replacement which carries a significant risk of morbidity and mortality. Reoperation is the standard of care and may still be required after the first successful surgery due to complications disrupting either mechanical or bioprosthetic valves. Such complications can be delayed or even prevented if optimal prosthesis selection is individualized according to patients' medical and postimplantation follow-up. We present the case of an 84-year-old woman where an open-heart valve-in-valve approach, implanting a mechanical valve in a failed bioprosthetic valve, produced a unique image on transthoracic echocardiography which needs to be recognized by imagers for appropriate patient diagnosis and management.

## 1. Introduction

Most bioprosthetic heart valves degenerate over a given time and therefore require repeat valve replacement. “Valve-in-valve” implantations, especially using a transcatheter approach, are now being encountered more frequently in daily clinical practice. Repeating open-heart surgery for heart valve replacement carries a significant risk of morbidity and mortality [1, 2]. Transcatheter heart valve implantation within a failed bioprosthesis, also known as the “valve-in-valve” procedure, is now more commonly performed thanks to the availability of percutaneous bioprosthetic valves helping patients outlive that same valve that prolonged their lives. Few reports exist in the literature of patients undergoing a valve-in-valve procedure for degenerative prostheses [3–6]. The key diagnostic tool of high-quality medical imaging, especially utilizing echocardiography, is at the center of identification of patients for appropriate guidance with respect to management. Echocardiography allows for adequate noninvasive evaluation through ever-advancing technology applications. We present the case of a valve-in-valve approach where a mechanical mitral valve was implanted in a failed bioprosthetic valve producing a unique image on transthoracic echocardiography.

## 2. Case Summary

An 84-year-old Black female presented with a chief complaint of chest congestion, dizziness, and malaise for two weeks. The patient had a past medical history significant for subacute bacterial endocarditis and status post porcine mitral valve replacement in 1984, followed by mechanical valve replacement in 2002 for which she was maintained on anticoagulation. The patient also had a history of nonischemic cardiomyopathy, nonobstructive coronary artery disease, hypertension, dyslipidemia, and type 2 diabetes mellitus. The patient was treated and discharged 15 days prior for abdominal pain likely secondary to viral gastroenteritis. Review of systems was otherwise negative. The patient denied any history of cigarette smoking, alcohol consumption, or recreational drug use. On physical exam, temperature was 97.2°F, respiratory rate was 18 per minute, blood pressure was 152/79 mmHg, heart rate was 69 beats per minute, and oxygen saturation was 98% on room air. The cardiac exam was significant for a mechanical valve S1, normal S2, regular rate and rhythm, and a II/VI soft blowing systolic murmur with maximum intensity best heard at the apex. Chest auscultation elicited diffuse expiratory rhonchi. Chest X-ray showed sternotomy wires and clear lungs. Chest CT showed no evidence for pneumonia or pulmonary edema. Electrocardiography (EKG) showed sinus rhythm at 64 beats per minute with first degree atrioventricular block and *Q* waves in leads V1 and V2 unchanged from a previous EKG. Laboratory results were significant for a supratherapeutic INR (4.5) and a negative set of cardiac markers. Coumadin was held, and she was admitted to the telemetry floor for continuous cardiac monitoring to rule out acute coronary syndrome.

**Figure 1:**
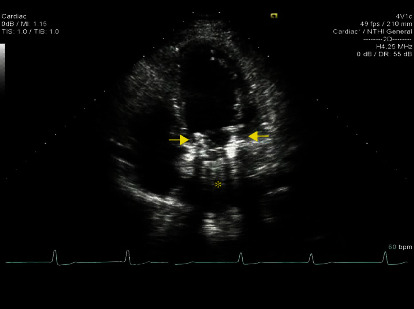
Apical four chamber transthoracic echocardiogram image of the mechanical valve (asterisk) implanted in the failed bioprosthetic valve (arrows).

Transthoracic echocardiography (TTE, Figure 1/Video 1) was performed to rule out structural causes of dizziness. Echocardiography revealed a mechanical mitral valve seen within a bioprosthetic porcine mitral valve with minimal mitral regurgitation. Mean transmitral valve gradient was 5 mmHg. Echo findings included the following: normal aortic root, calcified trileaflet aortic valve with normal opening, mild aortic regurgitation, and moderate segmental left ventricular systolic dysfunction with an ejection fraction (EF) of 45%. The basal to mid inferoseptal and inferolateral walls were severely hypokinetic, and the inferior wall appeared dyskinetic. The right ventricle appeared normal in size and function. The tricuspid valve was also normal but with mild-moderate tricuspid regurgitation. A left-sided heart catherization with ventriculography showed moderate diaphragmatic hypokinesis and severe posterobasal hypokinesis. Global left ventricular function was depressed, with the EF estimated by contrast ventriculography at 48%. The coronary circulation was right dominant. The left main coronary artery was normal. The mid-left anterior descending artery demonstrated a 40% tubular stenosis. The circumflex coronary artery showed minor luminal irregularities with no flow limiting lesions, and the proximal right coronary artery demonstrated a tubular 20% stenosis, while the mid-right coronary artery had a tubular 40% stenosis. There were no coronary lesions to account for the inferior wall motion abnormality.

The patient was restarted on warfarin after the INR became therapeutic and was discharged shortly after on medical management.

## 3. Discussion

The most common complication associated with bioprosthetic valves is structural valve deterioration. The incidence of structural failure with currently available porcine valves starts to increase 8 years after the operation and reaches over 60% at 15 years. It is noteworthy that the rate of failure of valves for those 70 years of age or older has been shown to be remarkably less than in younger age groups. Valvular limited durability is a major problem to long-term success of these bioprostheses [2]. The predominant causes of structural valve deterioration for porcine bioprostheses seem to be either calcification of the cusp tissue leading to mitral stenosis [5, 6] or leaflet tear leading to mitral regurgitation. Either one of these causes may necessitate reoperation.

In one study, the estimated overall mortality is around 12.5% for primary tissue failure of porcine bioprostheses; the study included both aortic and mitral valve replacement. Structural valve deterioration leading to reoperation is the cause for at least two thirds of the reoperations in patients with bioprostheses. Freedom from failure for all valvular locations (aortic, pulmonic, tricuspid, and mitral) of bioprosthetic valves at 10 years is estimated to be between 70-90% and 40-70% at 15 years. With current optimal medical management, the reported cases of degenerated valves are very rare for mechanical bileaflet, tilting disk, and ball-and-cage valves [2].

Risk factors for structural valve deterioration can be divided into two categories: patient-related factors and valve-related factors. Patient-related factors include age of the patient at the time of valve replacement and site of valve implantation. The rate of bioprosthesis failure at 10 years is less than 10% for those over 70 years of age, but for those younger, the rate of failure is 20-30%. Lifetime risk of reoperation decreases with increasing age of the patient. There have also been studies illustrating greater structural failure with valve replacement in the mitral versus aortic position secondary to the higher mechanical stress imposed on mitral valves during systole. Younger age, mitral valve position, renal injury, hyperparathyroidism, hypertension, left ventricular hypertrophy, reduced left ventricular function, and prosthesis size have been determined predictors of structural valve degeneration. In valve-related factors, new generation bioprostheses seem to be more durable than past bioprosthetic valves. There is also a suggestion that since bioprosthetic valves are fixated with glutaraldehyde, to reduce antigenicity and prevent extracellular remodeling, this biochemical makeup causes a calcium influx from membrane damage, promoting accelerated bioprosthetic tissue deterioration by providing an environment prone to calcium crystal accumulation and growth. Other studies document that in addition to a passive degenerative process, there may also be an active process of immune rejection and atherosclerosis resulting in bioprosthetic valve deterioration. There may be a component of humoral and/or cellular responses to animal tissue of replacement valves. In addition, as in native valves, atherosclerosis can destroy valves by inciting the inflammatory process. Several atherosclerotic risk factors, including hypercholesterolemia, diabetes, metabolic syndrome, and smoking, have been associated with structural valve degeneration. Therefore, it may not be a surprise to find that some studies have shown that statins have been associated with slowing the progression of structural valve deterioration [7, 8].

One major consideration in reoperation is increased mortality. However, there is ongoing debate about whether reoperation via open heart surgery even has an incremental effect to mortality compared with the primary operation and what risk factors are contributing to this mortality [4]. Many studies on mortality in reoperation are based on a heterogeneous group of patients who differ in factors such as their initial valve operation and factors leading to reoperation. Based on one report, the documented mortality rate of bioprosthetic rereplacement for structural valve deterioration ranges from 4% to 20% in most series, depending on risk factors and patient status [9, 10]. A number of studies evaluated mortality in reoperation as well as risk factors in determining those that are at high risk. However, the majority are neither comprehensive nor large in sample size. One study identified advanced age, pulmonary disease, cognitive impairment, higher NYHA functional class, reduced ejection fraction, renal disease, multiple reoperation, coronary artery bypass graft (CABG) at time of previous valve operation, or those who require concurrent CABG at valve reoperation, as conferring increased risk of death at reoperation [4, 9]. Also, the indication for reoperation, such as thrombosed valves or prosthetic valve endocarditis, also increases the risk of reoperation. On the other hand, this study found that greater caution is needed in patients requiring replacement of a mechanical valve in comparison to a bioprosthetic valve. Consideration is also needed for the fact that the risk of reoperation has also improved over time due to new techniques and advanced prostheses [4].

In those with high STS (Society of Thoracic Surgeons) risk scores and indications for repeat valve replacement; transcatheter heart valve (THV) implantation is becoming more mainstream compared to conventional open heart valve replacement. However, there needs to be better understanding and techniques of imaging to analyze these valve-in-valve prostheses as well as having the knowledge of post valve-in-valve echocardiographic parameters and hemodynamics to aid in better management of these patients. There also needs to be more comprehensive research on successful methods of percutaneous valve-in-valve implantation (such as coaxial positioning within the valve determined by perfected C-arm angulation, fixation of the secondary valve to the sewing ring of the bioprosthetic valve, and rapid ventricular pacing to minimize movement during deployment of the secondary valve), defining selection criteria for those that may not be suitable for transcatheter valve replacement (as in paravalvular regurgitation) and identification of valve-in-valve replacement issues (for example, incomplete leaflet coaptation due to an oversized and deformed secondary valve or a valve-in-valve leaflet “stuck” in an open position). Results have shown promise. One study showed that mitral valve-in-valve implantation reduced the mean gradient from 12.9 to 8.0 mmHg and increased the area from 0.7 to 1.7 cm^2^. While not statistically significant, the gradient was reduced or remained the same in all patients. In patients with severe stenosis as a major cause of valve failure, the mean gradient decreased from 18.3 to 7.3 mmHg, and in patients with mitral regurgitation as the major cause of valve failure, none had more than mild postprocedural regurgitation [1].

Often, the internal dimensions of the bioprosthetic valve restrict percutaneous valve expansion during valve-in-valve replacement, specifically by the relatively stiff external bioprosthetic valve ring (and at times by severe calcification of bioprosthetic valve leaflets). While on one hand, this decreases the probability of annular rupture, heart block, and coronary occlusion; on the other hand, valve underexpansion may contribute to hemodynamically significant residual gradients after valve-in-valve implantation. Theoretically, valve under expansion will worsen transvalvular gradients, effective orifice areas, and leaflet and stent durability. There are currently few long-term follow-up studies that have examined the clinical outcomes of such residual gradients. Valve-in-valve designs that reduce these residual gradients will become essential as younger and lower risk patients are treated in the future.

Manufacturers will also need to take into consideration ways to improve sizing in anticipation of possible future valve-in-valve replacements and the consideration for individual customization [1]. Although paravalvular regurgitation leaks are common after transcatheter aortic valve replacement for native aortic stenosis, regurgitation appears to be absent or mild in most published valve-in-valve reports. One suggestion is that the circular sewing ring of the bioprosthesis appears to facilitate intervalvular sealing. There are three sources of regurgitation in the setting of valve-in-valve management: these include paravalvular, intervalvular, and transvalvular leaks. There is also concern for coronary artery occlusion in aortic positions in failed bioprostheses with externally mounted leaflets or stentless valves, by valve-in-valve implantation. Although there are few studies on the durability of valve-in-valve replacements, midterm clinical data has shown that durability is sufficient to achieve significant clinical benefit in high-risk patients [1].

## 4. Conclusion

When bioprosthetic valves degenerate, reoperative valve replacement is still the current standard of care [9–12]. Transcatheter mitral valve replacement within a failed bioprosthetic valve is becoming a new viable option of secondary valve replacement in selected patients at high risk for open heart surgery/reoperation [6, 7]. Currently, valve-in-valve replacement has been successfully performed in aortic, mitral, pulmonic, and tricuspid bioprostheses [8, 9]. More studies are needed to evaluate long-term follow-up and outcomes, including quality of life of these percutaneous valve-in-valve replacement patients. Appropriate imaging and recognition of findings on imaging are required for adequate patient management and treatment.

## Data Availability

Our submission is a case report and therefore, the data used to support the findings of this case report are included within the article. No extra data is available as this is a case report.

## Supplementary Materials

Supplementary Materials Video 1: Apical four chamber transthoracic echocardiogram of the mechanical valve implanted in the failed bioprosthetic valve.
